# Hereditary analysis and genotype × environment interaction effects on growth and yield components of Bambara groundnut (*Vigna subterranea (L.) Verdc.*) over multi-environments

**DOI:** 10.1038/s41598-022-19003-z

**Published:** 2022-09-19

**Authors:** Md Mahmudul Hasan Khan, Mohd Y. Rafii, Shairul Izan Ramlee, Mashitah Jusoh, Md Al Mamun

**Affiliations:** 1 Laboratory of Climate-Smart Food Crop Production, Institute of Tropical Agriculture and Food Security (ITAFoS), Universiti Putra Malaysia (UPM), UPM Serdang, 43400 Selangor, Malaysia; 2 Department of Crop Science, Faculty of Agriculture, Universiti Putra Malaysia (UPM), 43400 Selangor, Malaysia; 3 Bangladesh Agricultural Research Institute (BARI), Gazipur, 1701 Bangladesh

**Keywords:** Plant sciences, Plant breeding

## Abstract

This investigation was carried out to explore G × E interaction for yield and its associated attributes in 30 Bambara groundnut genotypes across four environments in tropical Malaysia. Such evaluations are essential when the breeding program's objective is to choose genotypes with broad adaption and yield potential. Studies of trait relationships, variance components, mean performance, and genetic linkage are needed by breeders when designing, evaluating, and developing selection criteria for improving desired characteristics in breeding programs. The evaluation of breeding lines of Bambara groundnut for high yield across a wide range of environments is important for long-term production and food security. Each site's experiment employed a randomized complete block design with three replicates. Data on vegetative and yield component attributes were recorded. The analysis of variance revealed that there were highly significant (p ≤ 0.01) differences among the 30 genotypes for all variables evaluated. A highly significant and positive correlation was identified between yield per hectare and dry seed weight (0.940), hundred seed weight (0.844), fresh pod weight (0.832), and total pod weight (0.750); the estimated correlation between dry weight of pods and seed yield was 1.0. The environment was more important than genotype and G × E in determining yield and yield components.A total of 49% variation is covered by PC1 (33.9%) and PC2 (15.1%) and the genotypes formed five distinct clusters based on Ward hierarchical clustering (WHC) method. The genotypes S5G1, S5G3, S5G5, S5G6, S5G8, S5G7, S5G2, S5G4, S5G10, S5G13, S5G11, and S5G14 of clusters I, II, and III were closest to the ideal genotype with superior yield across the environments. The PCA variable loadings revealed that an index based on dry pod weight, hundred seed weight, number of total pods and fresh pod weight could be used as a selection criteria to improve seed yield of Bambara groundnut.

## Introduction

*Vigna subterranea* L. (Verdc), Bambara groundnut (2n = 2x = 22) is a legume crop in the Fabaceae family and subfamily Faboideae of the genus *Vigna* that has received little attention^1^. It first appeared in West Africa and is now a common food crop in African countries. It is also grown successfully in Asia and South Asia, including Malaysia, Thailand, India, the Philippines, Sri Lanka, India, and Brazil^2^. Bambara groundnut is the third most common legume in Africa after groundnut and cowpea since it can withstand drought and can be grown in low fertility soils where other crops fail^3^. By fixing nitrogen, it helps to increase soil fertility^4^. Surplus Bambara groundnuts are often sold in local markets, raising income for resource-limited farmers^5^. Bambara groundnut is a reliable source of food for low-income people^6^. The world's population is continuing to rise at an alarming pace, necessitating an increase in the production of this critical crop to counter potential demographic threats. In Malaysia, average production was 1.18 t ha^−1^^7^, 0.38 to 1.6 t ha^−1^^8^, and 0.97 to 3.41 t ha^−1^^9^ whereas 0.7 to 2.0 t ha^−1^^10^ was produced in Indonesia. At optimal farming conditions, it has the ability to produce up to 4.0 t ha^−1^^11^ and 5.0 t ha^−1^^3^ of the dry pod.

Scientists have investigated many ways to increase yields. These include selecting high-yielding varieties well adapted to particular growing areas, quality seed, crop establishment, nursery management, weed management, and post-harvest management. Breeding for high yield has been identified as the most sustainable approach since yield traits have heritability. However, there is high genotype by environment (GE) interaction for grain yield and more information is needed to identify broadly adapted high yielding genotypes. The lack of a modern production system and commercial high-yielding cultivars is the major limitation for this crop expansion; many growers still use traditional landrace varieties^12^. One of the main constraints to Bambara groundnut production in Malaysia is a scarcity of high-yielding cultivars. Plant breeders have used both conventional and molecular approaches to produce improved Bambara groundnut varieties^1^. Plant breeders routinely evaluate crop cultivars in broad environment tests using yield and its associated traits, as well as phenotypic expression. The variability in yield over environments (seasons and locations) is related to both biotic and abiotic environmental factors^13^.

In most crops, genotype by environment (G × E) interactions are common^14^ as certain genotypes have adapted to particular environments. A G × E interaction is characterized by a difference in the relative output of specific traits of two or more genotypes evaluated in two or more environments. This interaction usually changes genotype ranking across environments^15^. The uncertainty in identifying the target population in ecosystems (TPE), the lack of suitable selection criteria and finding suitable test locations to represent the target environments are all exacerbated by G × E interaction. Therefore, the focus on Bambara groundnut breeding programs has changed from developing genotypes with general adaption to identifying genotypes suited for particular conditions. Higher Bambara grain yield has the potential to improve food security. To discover superior and stable Bambara groundnut genotypes that are suited to several environments, the potential lines must be evaluated across those environments.

In order to assess G × E interaction in a multi-environmental yield trial, analysis of variance (ANOVA) is used in this study to test for differences between genotype, location and environment. Among those who have contributed to this work are Zobel et al.^16^ and Oladosu et al.^15^. Priority should be given to crop management and agronomical issues, especially during the vegetative stage and harvesting, according to Vadiveloo and Phang^17^, since improved lines generally require better growing conditions to achieve their yield potential. While the focus of any breeding program is to improve yield and grain quality, the identification of correlated traits that contribute to yield stability is important since yield is the net result of many plant processes. The current study aims to evaluate the contribution of several yield-related traits to yield stability in high yielding cultivars across four environments.

## Materials and methods

### Plant materials

The research work was conducted with the Institute of Tropical Agriculture and Food Security (ITAFoS), University Putra Malaysia (UPM), Malaysia. A set of 30 accessions of *V. subterranea* were used in this study. Initially, we collected 15 landrace seed samples from Nigeria from which we obtained 150 plants. These were selfed for 3 generations and then evaluated^6–8^. From that evaluation, we selected 44 lines which we selfed for 2 more generations and evaluated. We advanced the best 30 of these representing 11 of the 15 original accessions to this experiment. In terms of plant guidelines, we complied with relevant institutional, national, and international guidelines and legislation**.** We collected the plant seeds or specimens with the proper permission of the institution's authority by following the national and international strategies and deposited them in GenBank, ITAFoS, UPM. We also had appropriate permission from farm or field owners during collection and experimentation. We affirm that during the collection and execution of the experiment, the authors complied with the IUCN Statement on Research Involving Species at Risk of Extinction and the Convention on the Trade in Endangered Species of Wild Fauna and Flora. The name and ID of each accession are listed in Table 1.

**Table 1:** The list of thirty selected Bambara groundnut accession used in this study.

Genotype	ID	Genotype	ID	Genotype	ID
Maik12-18	S5G1	GiiwP12-18	S5G11	GiiwP9-18	S5G21
MaikP3-18	S5G2	ExSokP4-18	S5G12	GiiwP11-18	S5G22
MaikP6-18	S5G3	KarP10-18	S5G13	KarP8-18	S5G23
BdilaP5-18	S5G4	MaikP11-18	S5G14	DunP6-18	S5G24
JataP1-18	S5G5	MaibP8-18	S5G15	GiiwP1-18	S5G25
DunP9-18	S5G6	MaibP6-18	S5G16	KataP5-18	S5G26
CancP3-18	S5G7	KataP8-18	S5G17	KarP9-18	S5G27
RokP1-18	S5G8	DunP2-18	S5G18	DunP8-18	S5G28
ExSokP5-18	S5G9	CancP2-18	S5G19	RokP9-18	S5G29
ExSokP3-18	S5G10	BdilaP8-18	S5G20	JataP3-18	S5G30

The 11 accessions were: *Maik* Maikai, *Bdila* Bidillali, *Jata* Jatau, *Dun* Duna, *Canc* Cancaraki, *Rok* Roko, *ExSok* Exsokoto, *Giiw* Giiwa, *Kar* Karu, *Maib* Maibergo, *Kata* Katawa.

### Environment and location

Four field trials were conducted in two nearby fields in two cropping seasons (2020 and 2021) in Malaysia. These four environments represented a range of conditions in temperature, rainfall, soil type, soil structure, soil pH and management practices. Details of the environmental conditions were presented in Table 2. The soil properties of the experimental site are listed in Table 3.

**Table 2:** Environmental description of the experimental site.

Code	Season	Latitude	Longitude	Altitude	Av. temp	Av. hum (%)	Rainfall (mm)	Year
FTM (ENV 1)	Main	2.990935	101.7138	61.0 m	23.14 °C–29.88 °C	83.2	188.6	2020
FTO (ENV 2)	Off	2.990935	101.7138	61.0 m	24.22 °C–30.72 °C	82.6	198.4	2021
FFM (ENV 3)	Main	2.983092	101.7152	54.0 m	23.14 °C–29.88 °C	83.2	188.6	2020
FFO (ENV 4)	Off	2.983092	101.7152	54.0 m	24.22 °C–30.72 °C	82.6	198.4	2021

*FTM* Field ten main season, *FTO* Field ten off season, *FFM* Field fifteen main season, *FFO* Field fifteen off season, *ENV.* Environment, *Main season* May–September, *Off season* November–March, *Av. Temp.* Average temperature, *Av. Hum.* Average humidity. Sources: https://en.climate-data.org/asia/malaysia/selangor/mardi-serdang-971613/#climate-table.

**Table 3:** Characterization of soil properties of the experimental region.

Determination	Field fifteen (FF)	Field ten (FT)
Physical analysis	Value	
Sand (%)	40	5.8
Silt (%)	26.82	51.19
Clay (%)	33.74	42.99
Textural classes (USDA)	Clay loam	Silty clay
Chemical analysis	Value	
pH	6.6–7.5	5.0–5.59
Organic matter (%)	1.97	10.32
Total nitrogen (%)	0.16	0.41
Available phosphorus (mg kg^−1^)	10.6	59.2
Available potassium (mg kg^−1^)	120.6	306.4

### Experimental design and intercultural practices

The experiment was set up as a randomized complete block design (RCBD) with three replications in each environment. The experimental plot consisted of two rows 1.6 m × 0.80 m each. According to Khan et al.^8^, the distance between plants was 30 cm, row to row was 50 cm, plot to plot was 1.5 m, and the distance between replication was 2.0 m. Recommended intercultural activities such as field planning, land clearing, weeding, irrigation, and fertilizing were used during the growing season. The prescribed fertilizer rates (100% N = 45 kg N/ha, 100% P = 54 kg P_2_O_5_/ha, 100% K = 45 kg K_2_O/ha) and all portions of phosphorus and potassium were applied during final land preparation, though, 70% N was added at five weeks after planting^18^. The field was ploughed following the usual cultural traditions of the local farmers. Where necessary, pest and disease control was carried out. Regular hand weeding was conducted as needed.

### Data collection

According to Bambara groundnut description and descriptors by IPGRI, IITA, BAMNET^19^ twenty-seven numerical traits (Table 4) were assessed during data collection. The data were recorded from 5 randomly selected plants of each plot in each replication at several growth stages in the field and post-harvest data in the plant physiology lab.

**Table 4:** Twenty-seven quantitative traits measured according to IPGRI, IITA, BAMNET^19^.

Sl. no.	Quantitative traits	Code	Procedure of assessment
**Vegetative traits**			
1	Days to emergence	DTE (d)	Days between planting and the appearance of the first typical leaf
2	Days to 50% flowering	D50%F (d)	Seed germination to the arrival of 50% flowering
3	Days to maturity	DTM (d)	From the time of sowing through the first harvest
4	Plant height	PH (cm)	From the soil level to the tip of the topmost terminal leaflet
5	No. of branches/stem	NB	Data recorded immediately after harvest
6	No. of stems/plant	NS	Data recorded immediately after harvest
7	No. of petioles/plant	NP	Data recorded immediately after harvest
8	No. of leaves/plant	NL	Data recorded immediately after harvest
9	No. of nodes per stem	NNS	Data recorded immediately after harvest
10	Internode length	IL (cm)	Data recorded immediately after harvest
11	Biomass fresh weight/plant	BFW(g)	During harvesting, data was recorded
12	Biomass dry weight/plant	BDW(g)	Harvested plant dried in sun & data recorded
**Yield traits**			
13	Total no. of pods/plant	TNP	Data was counted at the time of harvesting
14	No. of mature pods	NMP	Data was counted at the time of harvesting
15	No. of immature pods/plant	NIP	Data was counted at the time of harvesting
16	Fresh pod weight	FPW (g)	Data was counted at the time of harvesting
17	Dry pod weight	DPW(g)	Harvested pods dried (12% moisture) in sun and recorded data
18	Pod length	PL (mm)	Measured the pod length using Digital Vernier Calliper
19	Pod width	PW (mm)	Measured the pod width using Digital Vernier Calliper
20	No. of seeds/plant	NSP	Data recorded after removing the shell of dried pods
21	Dry seed weight/plant	DSW(g)	Seeds dried (12% moisture) in sun and recorded data
22	Seed length	SL (mm)	Measured the seed length using Digital Vernier Calliper
23	Seed width	SW (mm)	Measured the seed width using Digital Vernier Calliper
24	100 seed weight	HSW (g)	100 dried seeds (12% moisture) counted and measured
25	Shelling percent (%)	Shell%	The ratio of dry seed and dry pod weight (12% moisture)
26	Harvest Index	HI (%)	Ratio of Grain yield (kg/ha.) / Biological yield (grain + straw)
27	Yield Kg per hectare	Yld (Kg/ha)	Dried pods (12% moisture) weight per plot converted to kg/ha

### Statistical analysis

#### Analysis of variance (ANOVA)

While data was collected on a randomly selected plant basis, it was analysed on a plot mean basis. Standard analyses of variance (ANOVA) were performed on the data using SAS version 9.4. The sources of variance tested were genotype (G), environment (E), and genotype by environment (G × E interaction). For each attribute, the mean, range, coefficient of variation (CV) and standard deviation were computed. Mean comparisons were carried out with the least significant difference in consideration (LSD) at 5% probability. Finally, the Pearson correlation was calculated on genotype mean basis using the means reported in Table 8 to study the correlations among the traits. The correlations between the quantitative variables were determined based on the rules given by Pearson^20^ using XLSTAT. Table 5 contains the ANOVA table for the expected mean squares for pooled locations and seasons or environments.

**Table 5:** Sketch of ANOVA table and EMS for pooled locations and seasons.

Source of variation	df	EMS
Rep (environment)	E(r − 1)	σ^2^_e_ + gσ^2^_s/r_
Environment (E)	(E − 1)	σ^2^_e_ + rσ^2^_gE_ + σ^2^_E_
Genotypes (G)	(g − 1)	σ^2^_e_ + rσ^2^_gE_ + σ^2^_g_
G × E	(g − 1) (E − 1)	σ^2^_e_ + rσ^2^_gE_
Error (E)	(r − 1) (gE − 1)	σ^2^_e_

*EMS* expected mean squares, *df* degree of freedom, *r* number of replication (3), *g* number of genotype (30), *E* number of environment (4).

The variance components were derived from the expected mean squares in Table 5 using “SAS proc varcomp” with a restricted maximum likelihood (REML) approach. The phenotypic variance was computed as follows. Phenotypic variance: σ^2^_p_ = σ^2^_g_ + σ^2^_gE_ + σ^2^_e._ where: σ^2^_g_ is the genotypic variance, σ^2^_gE_ is the G × E variance, and σ^2^_e_ is the mean error variance.

#### Estimation of variance components, heritability, and genetic advance

##### Phenotypic and genotypic coefficient of variation

According to Singh and Chaudhary^21^, the estimations of phenotypic and genotypic coefficients of variation were derived as follows:

$$(\mathrm{a})\mathrm{ PCV }\left(\mathrm{\%}\right)=\frac{\sqrt{{\upsigma }_{\mathrm{p}}^{2}}}{\overline{\mathrm{X}}} \times 100 \,\,\,\,\,\,\,\,\,\,\, (\mathrm{ b })\mathrm{ GCV }(\mathrm{\%})=\frac{\sqrt{{\upsigma }_{\mathrm{g}}^{2}}}{\overline{\mathrm{X}} } \times 100$$

where: PCV = Phenotypic coefficient of variation; GCV = Genotypic coefficient of variation; $\overline{\mathrm{X} }$ = Grand average of the characteristics; ${\upsigma }_{\mathrm{p}}^{2}$ = Phenotypic variance; ${\upsigma }_{\mathrm{g}}^{2}$ = Genotypic variance. According to Sivasubramanian and Madhava^22^, GCV and PCV levels were classified as low (0–10%), moderate (10–20%), and high (≥ 20%).

##### Heritability

The ratio of genotypic variation (${\upsigma }_{\mathrm{g}}^{2}$) to phenotypic variation (${\upsigma }_{\mathrm{p}}^{2}$) is defined as broad-sense heritability (${h}_{\mathrm{b}}^{2}$). Falconer^23^, defines the formula of heritability as follows:

$${h}_{b}^{2}\left(\%\right)=\frac{{\sigma }_{g}^{2}}{{ \sigma }_{p}^{2}} \times 100$$

where: The genotypic variance is denoted by ${\upsigma }_{\mathrm{g}}^{2}$, while the phenotypic variance is denoted by ${\upsigma }_{\mathrm{p}}^{2}$. According to Johnson et al.^24^ heritability percentages are classified as low (0–30%), moderate (30–60%), and high (≥ 60%).

##### Genetic advance

The genetic advance (GA) (as a percentage of the mean) was calculated using the Johnson et al.^24^ approach, with selection intensity (K) set to 5%. Following Johnson et al.^24^ the genetic advance was classified as modest (0–10%), moderate (10–20%), and high (> 20%).

$$GA\left(\%\right)=K\times \frac{\sqrt{{\sigma }_{p}^{2}}}{\overline{X}} \times {h }_{b}^{2} \times 100$$

where: K for constant also indicates the intensity of selection. According to Khan et al.^25^ the rate is 2.06 at the point when the K is at 5%. $\sqrt{{\upsigma }_{\mathrm{p}}^{2}}$= Standard deviation of phenotype; ${\mathrm{h}}_{\mathrm{b}}^{2}$ = Broad sense heritability and $\overline{\mathrm{X} }$ = Grand mean values of traits.

#### Multivariate analysis

To examine the relationships between the different variables in this study, the correlation coefficient was calculated using SAS software (version 9.4). The correlation heat map was generated using XLSTAT. To show the graphical relationship among principal axis, eigenvalues, and cumulative variance on a single plot, PCA variable, and case loading plot was created using XLSTAT. For two-way (double-dendrogram) clustering and constellation plot, we used JMP ver.16 software based on Ward’s hierarchical clustering (WHC) method. For scatter plot, density plot, and PCA 3D plot we followed NCSS 2021 program.

## Result

### Analysis of variance and mean performance

A greater understanding of the contribution of genotypes, environment, and their interaction as sources of heterogeneity is critical for developing more stable genotypes. The combined analysis of variance used for quantifying interactions and defining heterogeneity for agro-morphological traits indicated that the mean square for genotypes, environment and genotype × environment (G × E) demonstrated major variations at p ≤ 0.01 or p ≤ 0.05 or p > 0.05). The mean squares related to the G × E interactions of growth traits from a combined analysis of variance were summarised in Table 6. This investigation reflects the broad differences in genotype response to the environments for virtually all traits. Here, we focus on the traits significantly related to yield. The mean performance among the genotypes were displayed in Table 7.

**Table 6:** Mean square for growth and yield traits of 30 Bambara groundnut accessions revealed by ANOVA.

Trait	Rep (environment)	Environments	Genotypes	G × E	Error	CV	SD	Minimum (across replication)	Maximaum (across replication)
df	8	3	29	87	232				
**Vegetative traits**									
DTE	18.90**	18.34**	19.37**	5.33**	2.6	19.84	2.26	6.00	18.00
D50%F	170.93**	606.94**	124.08**	32.67**	5.11	14.09	5.49	27.00	58.76
DTM	186.58**	177.29**	265.12**	74.60**	10.46	5.43	7.20	110.00	156.00
PH	69.60**	663.21**	24.28**	13.63**	4.48	13.24	3.91	16.08	40.96
NB	122.61**	1668.60**	109.37**	51.20**	17.58	18.10	7.02	17.00	64.90
NS	120.58**	1151.56**	36.76**	21.08**	2.37	24.97	4.68	8.00	34.00
NP	1127.60*	20,927.79**	19,185.85**	9376.09**	469.73	21.67	65.77	153.47	432.30
NL	10,148.41*	188,350.13**	172,672.73**	84,384.88**	4227.61	21.67	197.31	460.41	1296.90
NNS	21.58**	15.74**	8.68**	6.41**	3.21	16.14	2.22	8.40	20.00
IL	3.74**	2.72**	0.98**	0.31**	0.18	17.61	0.62	2.24	5.34
BFW	14,799.31**	738,639.89**	103,871.17**	3875.17**	420.23	27.96	126.90	223.41	767.96
BDW	3321.27**	40,324.84**	47,005.4**	922.5**	183.73	26.75	67.46	147.99	480.38
**Yield traits**									
TNP	1042.51**	831.01**	220.44**	71.03**	19.44	10.41	8.82	51.90	106.50
NMP	877.07**	886.98**	234.05**	65.12**	18.07	12.11	8.56	42.90	91.30
NIP	36.55**	29.72**	36.50**	8.43**	3.93	20.96	2.93	8.00	22.00
FPW	9959.17**	428,488.77**	19,117.35**	6651.85**	256.82	13.32	84.41	289.64	810.22
DPW	4697.92**	191,292.95**	6228.69**	170.41*	127.54	11.98	47.40	311.53	530.87
PL	85.51**	466.32**	85.68**	54.33**	12.14	16.52	5.81	21.23	49.54
PW	68.87**	346.40**	8.58**	8.30**	2.5	16.36	2.96	9.70	30.89
NSP	1675.62**	517.20**	471.53**	38.32 ns	31.82	8.49	10.47	98.65	171.60
DSW	1918.50**	75,582.15**	4193.77**	420.84**	275.9	11.77	35.96	211.49	399.10
SL	85.44**	691.52**	10.82**	6.84**	2.16	19.62	3.41	10.15	26.32
SW	11.66**	124.57**	9.52**	5.95**	1.29	16.11	2.09	7.85	18.18
HSW	1886.90**	216,273.59**	1945.19**	204.52**	130.21	26.24	46.26	97.97	339.43
Shell%	73.88**	239.43**	31.95**	22.48 ns	17.34	6.18	4.78	54.58	94.31
HI	11.65**	424.48**	403.52**	4.28**	2.16	10.01	6.23	46.00	75.28
YLD	166,448.32**	6,423,330.79**	220,685.25**	6038.15*	4519.1	11.98	282.13	1854.37	3159.94

“**” correlation is significant at the 0.01 level; “*” correlation is significant at the 0.05 level, *SD* standard deviation, *CV* coefficient of variation (%), *DTE* (day) Days to emergence, Days to 50% flowering (day), *DTM* Days to maturity (day), *PH* Plant height (cm), *NB* Number of branches per plant, *NS* Number of stems per plant, *NP* Number of petioles per plant, *NL* Number of leaves per plant, *NNS* No. of nodes per stem, *IL* Inter nodes length (cm), *BFW* Biomass fresh weight per plant (g), *BDW* Biomass dry weight per plant (g), *TNP* Total no. of pods per plant, *NMP* Number of mature pods per plant, Number of Immature pods per plant, *FPW* Fresh pods weight (g), *DPW* Dry pods weight (g), *PL* Pod length (mm), *PW* Pod width (mm), *NSP* Number of seeds per plant, *DSW* Dry seed weight per Plant (g), *SL* Seed length (mm), *SW* Seed width (mm), *HSW* hundred seed weight (g), *Shell %* Shelling percent, *HI* Harvest index (%) and *Yld* Yield (Kg/ha).

**Table 7:** Means performance and comparison of 30 Bambara groundnut genotypes tested four environments.

Geno	DTE	D50%F	DTM	PH	NB	NS	NP	NL	NNS	IL	BFW	BDW	TNP	
G1	9.84a	32.75no	132.25d–g	29.74d–h	40.41b–g	19.42c–e	359.40ab	1078.22ab	14.5608a	3.84a–e	546.65a	314.93e	88.38b–f	
G2	9.55ab	32.61o	129.66g–j	30.62b–e	42.526a–c	17.09h–k	315.80ef	947.42ef	14.5092ab	3.43f–k	459.68a	259.85f	89.46a–c	
G3	9.77ab	34.45mn	132d–g	29.16e–i	41.08b–e	19.12d–f	229.29p	687.87p	13.3658ab	3.58c–g	582.89b	343.91b	89.24a–d	
G4	11.88a–c	37.50i–k	130.66f–i	28.20h–k	38.02e–i	19.88cd	353.87ab	1061.64ab	14.32a–c	3.86a–d	443.78b	245.87gh	92.26a	
G5	12.32a–d	35.77k–m	131.08e–h	30.47b–f	40.23b–g	17.54g–k	279.95h–k	839.87h–k	14.19a–d	3.53d–i	437.48b	241.22hi	87.93c–f	
G6	10.40a–e	36.78jk	136.33c	31.66a–c	38.37e–i	20.60bc	240.47op	721.41op	11.94a–e	3.55d–h	403.97b	221.27k–m	87.41c–g	
G7	10.85b–e	38.61hi	133.08d–f	27.02k	39.46c–g	19.87cd	252.8no	758.4no	14.80a–e	3.25g–k	456.87c	253.36fg	88.39b–f	
G8	12.51b–e	38.22h–j	132.5d–f	28.70g–k	33.312j	17.40h–k	327.35de	982.06de	14.68a–e	3.16jk	346.04c	180.61n	87.32c–g	
G9	13.14b–f	41.37e–d	140b	28.33h–k	36.01h–j	20.51bc	318.49ef	955.5ef	14.16a–f	3.18i–k	404.08d	219.88lm	87.56c–g	
G10	11.95b–g	46.21a	146.83a	28.80f–j	39.29c–h	17.91f–j	349.05bc	1047.16bc	13.5267a–f	3.40f–k	468.42d	261.81f	86.28c–h	
G11	10.58c–g	39.61f–h	128j	29.72d–h	39.03d–h	17.72g–j	293.49g–i	880.48g–i	12.71a–g	3.1503 k	425.01de	230.11j–l	91.88ab	
G12	13.79c–h	43.25bc	141.08b	27.60i–k	37.57f–i	20.59bc	254.50no	763.52no	12.86a–g	3.24g–k	325.82de	163.38o	80.09l–n	
G13	10.98c–h	39.61f–h	139.58b	31.29a–d	42.93ab	16.97i–k	369.05a	1107.18a	12.93a–h	3.54d–h	581.17de	339.24bc	85.56e–i	
G14	9.43d–i	34.72lm	129.66g–j	29.38e–h	40.93b–f	17.50g–k	301.08fg	903.25fg	14.11a–h	4.19a	425.38ef	232.07i–k	85.73d–i	
G15	13.33d–j	38.38h–j	139.5b	27.27jk	38.95d–h	19.50cd	257.02m–o	771.07m–o	13.16a–i	3.14 k	325.18fg	164.57o	84.12g–k	
G16	12.65d–j	41.97c–e	128.41ij	28.05h–k	37.23g–i	20.37bc	357.37ab	1072.12ab	15.31b–i	3.16jk	407.84fg	219.29lm	84.21g–k	
G17	12.05e–j	39.59g–i	134 cd	32.74a	40.02b–g	21.22b	261.27l–n	783.81l–n	14.49b–i	3.54d–h	647.17gh	384a	84.88f–k	
G18	9.08f–k	39.15g–i	132.58d–f	30.03c–g	38.83d–h	18.04f–i	343.82b–d	1031.48b–d	14.35c–i	3.67b–f	461.38gh	255.18fg	83.31h–l	
G19	10.62f–k	35.81k–m	132.58d–f	28.31h–k	39.88b–g	19.02d–f	272.69j–m	818.09j–m	12.47c–i	3.90a–c	392.92hi	212.14m	81.84j–n	
G20	12.33g–l	40.52e–g	134.08cd	30.57b–e	35.96h–j	17.89f–j	332.99c–e	998.99c–e	13.68c–j	3.22h–k	396.38ij	212.97m	88.81a–e	
G21	11.71g–m	38.60hi	133.66de	30.70b–e	37.94e–i	17.66g–j	325.08e	975.26e	13.98d–j	3.95ab	430.16i–k	234.60ij	85.15f–j	
G22	10.98h–n	39.40gh	129.33h–j	32.00ab	41.81b–d	16.34k	348.23bc	1044.71bc	13.30e–j	3.53d–i	327.26i–k	165.04o	82.41i–m	
G23	11.59i–n	36.28kl	129.75g–j	30.71be	38.46d–i	17.60g–j	278.30i–l	834.92i–l	12.78f–j	3.22h–k	391.01jk	214.74m	81.58k–n	
G24	9.66j–n	39.14g–i	127.16j	27.61i–k	29.725k	16.69jk	262.65k–n	787.96k–n	13.74f–j	3.31g–k	322.31jk	164.43o	73.05o	
G25	11.47k–o	43.01b–d	133.33de	30.03c–g	40.09b–g	24.68a	296.20gh	888.62gh	13.90f–j	3.50e–j	543.85k	312.89e	80.39l–n	
G26	11.90k–o	39.77f–h	127.75j	29.68d–h	45.87a	19.65cd	275.39j–l	826.2j–l	12.95 g–j	3.41f–k	458.61l	254.43fg	82.47i–m	
G27	11.22l–o	41.73c–e	128j	28.81f–j	39.04d–h	17.38h–k	294.11g–i	882.34g–i	13.47 g–j	3.84b–e	580.46m	332.66cd	79.70mn	
G28	10.80m–o	39.49gh	127.25j	29.46e–h	36.01h–j	18.70d–g	289.28g–j	867.86g–j	13.00 h–j	3.31g–k	635.36m	373.99a	82.60i–m	
G29	12.24no	39.84f–h	129.25h–j	29.22e–i	35.25ij	18.26e–h	323.6e	970.8e	15.01ij	3.73b–f	413.49m	229.55j–l	79.92l–n	
G30	13.3275o	44.605ab	133.33de	28.84f–j	39.51c–g	17.34h–k	344.42b–d	1033.28b–d	14.97j	3.84b–e	574.75m	327.34d	78.31n	
Mean	11.40 ± 0.11	38.96 ± 0.28	132.75 ± 0.37	29.49 ± 0.20	38.79 ± 0.37	18.75 ± 0.24	303.57 ± 3.46	910.71 ± 10.39	13.77 ± 0.11	3.51 ± 0.03	453.85 ± 6.68	252.18 ± 3.55	84.67 ± 0.46	
LSD	1.29	1.82	2.6	1.7	3.3731	1.2399	17.433	52.299	1.4422	0.3468	16.489	10.903	3.5468	
Max	13.79	46.21	146.83	32.74	45.873	24.685	369.059	1107.18	15.3167	4.1927	647.179	384	92.262	
Min	9.08	32.61	127.16	27.02	29.725	16.3467	229.291	687.87	11.9408	3.1494	322.31	163.387	73.05	

Geno	NMP	NIP	FPW	DPW	PL	PW	NSP	DSW	SL	SW	HSW	Shell%	HI	Yld
G1	76.83ab	11.55kl	675.71a–c	430.12a	40.312a	19.84a	131.71a–c	335.73a	18.90ab	15.09a	190.31ab	78.21a–e	58.84k	2560.29a
G2	78.25a	11.21l	681.56a	424.75ab	38.27a–c	18.51b–f	132.17ab	329.18ab	18.21a–e	13.89bc	191.69ab	77.77a–e	63.39fg	2528.33ab
G3	77.97ab	11.26l	684.58a	422.60a–c	38.63ab	18.59a–f	130.22a–d	322.90a–e	18.65a–c	14.23ab	182.91b–f	76.56b–f	56.22l	2515.5a–c
G4	77.53ab	14.72b–f	661.73d–f	425.04ab	37.50b–e	19.07a–e	133.91a	322.34b–e	17.50c–i	12.48f–k	185.44b–d	76.09d–f	63.91ef	2530.05ab
G5	74.77b–d	13.16f–j	666.89b–d	412.36d–f	37.57a–e	19.48a–c	128.94b–e	315.10c–h	18.21a–e	12.68e–j	197.16a	76.36b–f	63.72e–g	2454.53d–f
G6	71.99d–f	15.42a–e	663.27c–e	416.30b–d	35.03e–h	19.76ab	128.17b–f	322.66a–e	17.85b–h	13.45b–e	186.30bc	77.60a–f	65.45 cd	2477.99b–d
G7	75.01a–d	13.38f–j	676.35ab	413.84c–f	35.31d–h	19.19a–d	130.91a–d	315.90b–g	17.69c–i	12.95d–i	172.79gi	76.47b–f	62.66gh	2463.37c–f
G8	72.74c–e	14.58c–f	664.91b–d	414.94c–e	32.86h–l	18.42c–f	127.63c–f	326.41a–d	17.45d–i	12.22 h–l	188.21a–c	78.92a–e	69.73b	2469.92c–e
G9	71.24e–h	16.31ab	665.29b–d	412.38d–f	35.30d–h	17.75f–i	127.66b–f	327.76a–c	18.03a–f	12.66e–j	185.57b–d	79.63ab	65.80c	2454.7d–f
G10	71.70d–g	14.58c–f	663.74b–d	410.12d–g	32.99g–k	19.42a–c	126.63d–h	314.94c–h	18.17a–e	14.27ab	187.52bc	77.07b–f	61.62hi	2441.21d–g
G11	75.68a–c	16.19ab	649.59fg	406.93e–h	38.01a–d	18.25c–g	129.46a–d	301.96h–l	19.10a	12.98c–h	175.34e–h	74.31f	64.45d–f	2422.21e–h
G12	67.29i–l	12.79g–l	562.16lm	410.83d–f	33.06g–k	17.69f–i	126.88d–g	313.78d–h	17.20d–j	12.72e–j	191.75ab	76.21c–f	72.02a	2445.45d–f
G13	72.61c–e	12.95g–k	662.51de	405.37f–h	33.22g–k	17.81e–i	127.26c–f	317.71b–f	16.78 g–k	12.61e–j	182.66b–f	78.61a–e	55.46l	2412.93f–h
G14	72.19d–f	13.54f–i	650.49e–g	401.29g–i	36.68b–f	17.86e–h	122.64g–j	307.09f–k	17.06e–j	12.29 g–l	174.02f–i	76.437b–f	64.00ef	2388.67g–i
G15	72.19d–f	11.92j–l	658.95d–f	405.45fh	33.72g–k	16.56i	124.88e–h	308.73f–j	17.50c–i	12.94d–i	172.50 g–i	76.32b–f	71.35a	2413.4f–h
G16	69.86e–j	14.35d–g	635.88hi	388.99k–m	34.67f–i	16.98g–i	120.02i–k	308.94f–j	16.8g–k	12.99c–h	174.28f–i	79.31a–d	64.65c–e	2315.42k–m
G17	70.66e–i	14.21d–g	645.95gh	398.64h–j	33.48g–k	17.85e–h	124.38f–i	314.47c–h	18.32a–d	13.68b–d	185.39b–d	79.01a–e	52.29m	2372.86h–j
G18	70.02e–j	13.29f.–j	642.01gh	394.33i–k	33.74g–k	17.49f–i	122.67gj	297.15j–l	17.12e–j	12.33f–k	169.43h–j	75.68ef	61.52hi	2347.23i–k
G19	67.92h–k	13.91e–h	628.15i	394.62i–k	38.27a–c	17.58f–i	122.11h–j	311.13e–i	16.78g–k	11.61kl	176.95d–h	78.89a–e	65.50cd	2348.94i–k
G20	72.22d–f	16.58a	650.71e–g	391.45j–l	33.93f–j	18.12d–h	122.62g–j	299.01i–l	17.95a–g	12.06i–l	183.94b–e	76.80b–f	65.35cd	2330.09j–l
G21	69.03f–k	16.11a–c	624.27i	383.28l–n	38.79ab	17.46f–i	118.82j–l	299.13i–l	16.76h–k	13.19c–g	180.07c–g	78.14a–e	62.62gh	2281.46l–n
G22	70.51e–i	11.90j–l	607.48j	378.64no	34.45f–i	18.12d–h	117.32k–m	303.80g–k	17.51c–i	13.64b–d	169.64h–j	80.46a	70.05b	2253.82no
G23	66.27k–m	15.31a–e	604.18jk	381.23mn	38.05a–d	17.93d–h	117.03k–m	302.28h–l	16.72h–k	13.24c–f	176.94d–h	79.28a–d	64.40d–f	2269.25 mn
G24	61.06n	12.04i–l	593.95k	378.29no	30.08l	17.00g–i	119.28jk	289.35lm	15.84k	11.85j–l	161.51j	76.66b–f	69.72b	2251.74no
G25	68.49g–k	11.90j–l	636.66hi	372.05op	37.63a–e	17.78f–i	114.49l–n	294.84kl	17.76b–h	14.34ab	171.47g–i	79.54a–c	55.14l	2214.62op
G26	66.69j–m	15.78a–d	597.98jk	371.51op	35.75c–g	17.80e–i	116.32k–n	279.83mn	16.99f–k	13.43b–e	165.13ij	75.72ef	60.14j	2211.42op
G27	63.58mn	16.11a–c	558.78m	365.84qp	33.38g–k	17.48f–i	114.22mn	270.76no	16.75h–k	13.19c–g	150.42k	74.37f	53.47m	2177.63pq
G28	66.01k–m	16.58a	561.53lm	361.47q	31.18j–l	17.73f–i	115.83k–n	273.56no	16.52i–k	11.63kl	148.45k	75.71ef	50.57n	2151.62q
G29	64.37ln	15.55a–d	573.23l	350.76r	31.96i–l	18.22c–g	112.49n	277.03mn	14.58l	11.39l	160.97j	79.43a–d	60.50ij	2087.88r
G30	65.87k–m	12.43h–l	559.89m	343.57r	31.02kl	16.86hi	113.74mn	261.30o	16.12jk	12.13h–l	150.98k	78.34a–e	52.73m	2045.12r
Mean	70.68 ± 0.45	13.99 ± 0.15	633.61 ± 4.44	395.57 ± 2.49	35.16 ± 0.30	18.09 ± 0.15	123.35 ± 0.55	305.49 ± 1.89	17.36 ± 0.17	12.94 ± 0.10	176.32 ± 2.43	77.46 ± 0.25	62.24 ± 0.32	2354.59 ± 14.86
LSD	3.4195	1.5965	12.89	9.084	2.8026	1.2738	4.5375	13.361	1.1825	0.9151	9.1786	3.3495	1.1848	54.072
Max	78.256	16.5867	684.586	430.128	40.312	19.8424	133.919	335.731	19.1057	15.0942	197.163	80.464	72.0239	2560.29
Min	61.006	11.21	558.789	343.578	30.086	16.5601	112.494	261.308	14.5895	11.3941	148.456	74.316	50.5714	2045.12

*Geno* genotypes, *LSD* list significant difference, *DTE* Days to emergence (d), Days to 50% flowering (d), *DTM* Days to maturity (d), *PH* Plant height (cm), *NB* Number of branches per plant, *NS* Number of stems per plant, *NP* Number of petioles per plant, *NL* Number of leaves per plant, *NNS* No. of nodes per stem, *IL* Inter nodes length (cm), *BFW* Biomass fresh weight per plant (g), *BDW* Biomass dry weight per plant (g), *TNP* Total no. of pods per plant, *NMP* Number of mature pods per plant, *NIP* Number of Immature pods per plant, *FPW* Fresh pods weight (g), *DPW* Dry pods weight (g), *PL* Pod length (mm), *PW* Pod width (mm), *NSP* Number of seeds per plant, *DSW* Dry seed weight per Plant (g), *SL* Seed length (mm), *SW* Seed width (mm), *HSW* hundred seed weight (g), *Shell %* Shelling percent, *HI* Harvest index (%) and *Yld* Yield (Kg/ha).

#### Analysis of variance and mean performance for vegetative components

Days to emergence (DTE) differed significantly (p ≤ 0.01) among environments, genotypes and genotype by environment (G × E). The average emergence period spanned from 9 days (S5G18) to 13 days (S5G12) with an average of 11 days (Table 7). Days to 50% flowering (D50%F) showed highly significant variation (p ≤ 0.01) for all sources of variation. Genotype S5G10 took more time (46 days) to reach 50% flowering in the plant while the genotype S5G1 and S5G2 produced 50% flower in a relatively short time (32 days). However, the average days to flowering were recorded as 39 days after planting (Table 7). Days to maturity (DTM) differed among genotypes and environments and their interaction. The longest duration in days to maturity was observed in S5G10 at 132 days while the shortest days to maturity were observed in S5G11, S5G26, S5G28, and S5G24 at 127 days followed by S5G27 at 128 days as indicated in Table 7. Plant height (PH, cm) varied significantly (p ≤ 0.05) among the genotypes (G), environment (E), G × E (Table 6). The tallest genotype was S5G17 (32.74 cm) while the lower was 27.2 cm (S5G7) with an average of 29.49 cm as shown in Table 10. Biomass fresh weight (BFW, g) per plant was significantly (p ≤ 0.01) different for all sources of variation. The average weight (g) of fresh biomass was noted as 453 g, with a range of 322 g (S5G24) to 647 g (S5G17) followed by 635 (S5G28) as displayed in Table 7. Biomass dry weight (BDW, g) per plant was significantly (P ≤ 0.01) different for all sources of variation. The average weight of dry biomass was noted as 252 g, with a range of 163 g (S5G12) to 384 g (S5G17) followed by 373 g (S5G28) as displayed in Table 7.

#### Analysis of variance and mean performance for yield components

The number of pods per plant (TNP) was significantly (p ≤ 0.01) different for genotype (G), environment (E) and G × E. The average number of pods per plant was 84, with a range of 73 (S5G24) to 92 (S5G4) as displayed in Table 7. A highly significant difference (p ≤ 0.01) was observed for all sources of variations for the trait fresh pod weight (FPW). The average weight of fresh pods was 633.61 g, with a range of 558.78 g (S5G27) to 684.58 g (S5G3) as displayed in Table 7. Except for genotype by environment (G × E) all other sources showed a highly significant variation (p ≤ 0.01) for dry pod weight (g). The average weight of dry pods (DPW) was 395.57 g, with a range of 343.57 g (S5G30) to 430 g (S5G1) as indicated in Table 7. Genotype by environment (G × E) had significant variation at p ≤ 0.01 for dry seed weight (DSW, g). The average weight of dry seed was 305.49 g, with a range of 261.30 g (S5G30) to 335.73 g (S5G1) as presented in Table 7. For a hundred seed weight (g) (HSW) showed a highly significant variation (p ≤ 0.01) for genotypes, environments, and G × E.

The average hundred seed weight (g) was 176.32 g, with a range of 148.45 g (S5G28) to 197.16 g (S5G5) as indicated in Table 7. For shelling percentage (Shell%) all the source of variation showed highly significant (p ≤ 0.01). The average shelling percentage was 77%, with a range of 74% (S5G11) to 80% (S5G22) as shown in Table 7. For harvest index (HI %), genotypes, environments, and their interaction (G × E) were observed highly significant variation (p ≤ 0.01). The average values were 62%, with a range of 50% (S5G28) to 72% (S5G12) followed by S5G15 (71%) for harvest index as indicated in Table 7. The yield per hectare had highly significant difference (p ≤ 0.01) for genotype, environments, though a significant (p ≤ 0.05) variation has noted for interaction of genotype with environment. The average yield per hectare was 2354.59 kg/ha, with a range of 2045.12 kg/ha (S5G30) to 2560.29 kg/ha (S5G1) as specified in Table 7.

### Estimation of the relationship between traits

#### Correlation between growth and yield components

The correlations among overall trait means for the vegetative and yield components are shown in Table 8. Among the 27 traits we considered 12 as vegetative traits. Days to emergence showed negative and weak association with dry pod weight (r = − 0.19), dry seed weight (r = − 0.12), and yield (r = − 0.19) while positive and moderately significant correlation was found with harvest index (r = 0.16), days to maturity ( r = 0.40), and days to 50% flowering (r = 0.58). Days to 50% flowering showed positive and intermediate significant association with DTM (r = 0.38), whereas negative significant difference was recorded with total number of pods (r = − 0.40), dry pods weight (r = − 0.48), harvest index (r = − 0.13), and yield (r = − 0.48). Days to maturity had no meaningful association with plant height (r = − 0.09), though positive and significant association was noted with dry pods weight (r = 0.37), fresh pod weight (r = 0.29), hundred seed weight (r = 0.46), and yield (r = 0.37). Plant height had positive and significant association with number of branch (r = 38), hundred seed wieght (r = 0.20) though negative and non significant association was ovserved with dry pod weight (r = − 0.04), harvest index (r = − 0.30), and yield (r = − 0.04). Number of branches exhibited positive and significant correlation with biomass fresh and dry weight (r = 0.36) but there was no significant relation with yield (r = 0.13). There was no meaningful correlation of yield with number of petiole and leaves though perfect positive and highly significant correlation (r = 1.00) was found between number of petiole and leaves. Biomass fresh weight showed negative significant association with harvest index (r = − 0.97), though no significant variations was noted with fresh pod weight (r = − 0.03), dry pods weight (r = − 0.15), dry seed weight (r = − 0.20), and yield (r = − 0.15). However, harvest index (r = − 0.96) and hundred seed weight (r = − 0.22) had negative and moderately non significant correlation with biomass dry weight.

**Table 8:** Pearson's correlation (r) estimates for 27 phenotypes of 30 accessions of Bambara groundnut.

Trait	DTE	D50%F	DTM	PH	NB	NS	NP	NL	NNS	IL	BFW	BDW			
DTE	**1.000**	0.587**	0.401*	− 0.241	− 0.165	0.244	0.071	0.071	0.138	− 0.354*	− 0.198	− 0.209			
D50%F		**1.000**	0.389*	− 0.210	− 0.203	0.192	0.214	0.214	0.051	− 0.270	0.046	0.024			
DTM			**1.000**	− 0.093	0.051	0.223	0.063	0.063	− 0.167	− 0.167	− 0.072	− 0.072			
PH				**1.000**	0.382*	− 0.105	0.134	0.134	− 0.187	0.191	0.307	0.317			
NB					**1.000**	0.087	0.024	0.024	− 0.190	0.293	0.365*	0.363*			
NS						**1.000**	− 0.291	− 0.291	0.015	− 0.136	0.186	0.190			
NP							**1.000**	1.000	0.447*	0.207	0.065	0.055			
NL								**1.000**	0.447*	0.207	0.065	0.055			
NNS									**1.000**	0.136	0.130	0.126			
IL										**1.000**	0.318	0.313			
BFW											**1.000**	0.999**			
BDW												**1.000**			

Trait	TNP	NMP	NIP	FPW	DPW	PL	PW	NSP	DSW	SL	SW	HSW	Shel%	HI	Yld
DTE	− 0.113	− 0.211	0.258	− 0.266	− 0.196	− 0.309	− 0.256	− 0.170	− 0.129	− 0.155	− 0.175	0.068	0.220	0.161	− 0.196
D50%F	− 0.400*	− 0.496*	0.274	− 0.462*	− 0.483*	− 0.615**	− 0.363*	− 0.439*	− 0.477**	− 0.263	− 0.133	− 0.345	0.024	− 0.130	− 0.483**
DTM	0.148	0.179	− 0.089	0.296	0.370*	− 0.139	0.135	0.337	0.397*	0.253	0.206	0.466	0.145	0.156	0.370*
PH	0.163	0.128	0.078	0.117	− 0.045	0.191	0.228	− 0.084	0.062	0.224	0.307	0.204	0.299	− 0.301	− 0.045
NB	0.284	0.375*	− 0.251	0.218	0.139	0.473*	0.184	0.116	0.110	0.350*	0.515**	0.138	− 0.044	− 0.319	0.139
NS	0.049	0.061	− 0.032	0.136	0.113	0.192	0.055	0.051	0.176	0.236	0.319	0.148	0.163	− 0.160	0.113
NP	0.137	0.113	0.052	0.053	− 0.082	− 0.054	0.009	− 0.053	0.003	− 0.107	− 0.020	0.016	0.308	− 0.093	− 0.082
NL	0.137	0.113	0.052	0.053	− 0.082	− 0.054	0.009	− 0.053	0.003	− 0.107	− 0.020	0.016	0.308	− 0.093	− 0.082
NNS	0.056	0.127	− 0.185	0.124	− 0.052	− 0.087	− 0.018	0.008	0.014	− 0.124	− 0.044	− 0.011	0.259	− 0.147	− 0.052
IL	− 0.069	− 0.021	− 0.116	− 0.095	− 0.138	0.264	0.090	− 0.147	− 0.147	− 0.211	− 0.006	− 0.138	0.004	− 0.354*	− 0.138
BFW	0.073	0.072	− 0.005	− 0.037	− 0.158	0.020	0.088	− 0.085	− 0.204	0.133	0.273	− 0.237	− 0.109	− 0.970**	− 0.158
BDW	0.082	0.082	− 0.008	− 0.020	− 0.145	0.032	0.103	− 0.075	− 0.185	0.136	0.278	− 0.221	− 0.094	− 0.968**	− 0.145
TNP	**1**	0.920**	0.128	0.786**	0.750**	0.558**	0.621**	0.785**	0.670**	0.736**	0.303	0.636**	− 0.162	0.064	0.750**
NMP		**1**	− 0.271	0.862**	0.831**	0.592**	0.605**	0.857**	0.767**	0.783**	0.435**	0.688**	− 0.085	0.099	0.831**
NIP			**1**	− 0.250	− 0.262	− 0.128	− 0.007	− 0.240	− 0.295	− 0.174	− 0.358	− 0.181	− 0.181	− 0.093	− 0.262
FPW				**1**	0.832**	0.524**	0.529**	0.797**	0.832**	0.714**	0.413**	0.726**	0.074	0.186	0.832**
DPW					**1**	0.489*	0.587**	0.964**	0.940**	0.740**	0.393*	0.844**	− 0.121	0.364*	1.00**
PL						**1**	0.408*	0.408**	0.501**	0.552**	0.519**	0.499**	0.044	0.045	0.489**
PW							**1**	0.603**	0.550**	0.524**	0.417**	0.563**	− 0.054	− 0.005	0.587**
NSP								**1**	0.866**	0.712**	0.289	0.778**	− 0.200	0.288	0.964**
DSW									**1**	0.662**	0.418**	0.889**	0.218	0.392*	0.940**
SL										**1**	0.661**	0.641**	− 0.170	0.050	0.740**
SW											**1**	0.383*	0.105	− 0.168	0.393*
HSW												**1**	0.181	0.393**	0.844**
Shel%													**1**	0.067	− 0.121
HI														**1**	0.364*
Yld															**1**

“**”correlation is significant at the 0.01 level; “*” correlation is significant at the 0.05 level, Days to emergence = DTE (d), Days to 50% flowering (d), *DTM* Days to maturity (d), *PH* Plant height (cm), *NB* Number of branches per plant, *NS* Number of stems per plant, *NP* Number of petioles per plant, *NL* Number of leaves per plant, *NNS* No. of nodes per stem, *IL* Inter nodes length (cm), *BFW* Biomass fresh weight per plant (g), *BDW* Biomass dry weight per plant (g), *TNP* Total no. of pods per plant, *NMP* Number of mature pods per plant, *NIP* Number of Immature pods per plant, *FPW* Fresh pods weight (g), *DPW* Dry pods weight (g), *PL* Pod length (mm), *PW* Pod width (mm), *NSP* Number of seeds per plant, *DSW* Dry seed weight per Plant (g), *SL* Seed length (mm), *SW* Seed width (mm), *HSW* hundred seed weight (g), *Shell %* Shelling percent, *HI* Harvest index (%) and *Yld* Yield (Kg/ha).

#### Correlation between yield and yield components

**Figure 1:**
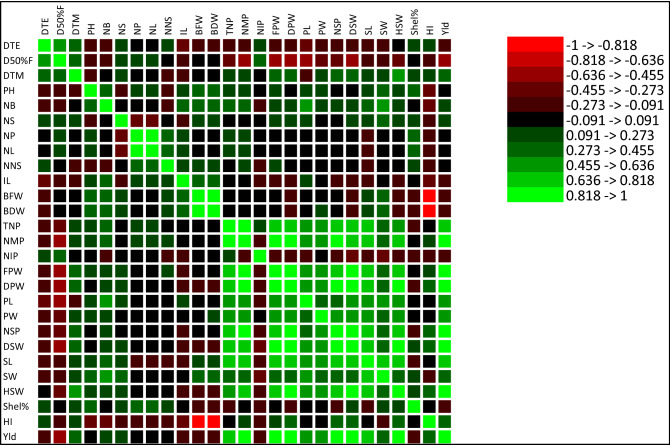
Correlation cluster heatmap showing graphical relationship among the 27 traits revealed by XLSTAT. Note: Red and yellow colour indicate negative and positive correlation, respectively among the tested traits. Darker the hue greater the relation between the traits and vice versa.

**Figure 2:**
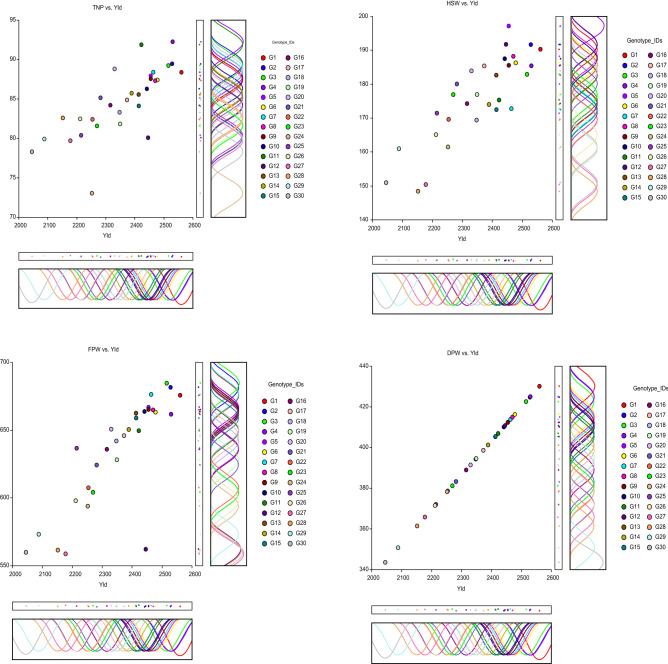
Scatter matrix with density and dot plot illustrates the graphical relationship of yield and its components in Bambara groundnut accessions revealed by NCSS 2021. (**A**) The total number of pods (TNP) vs Yield, (**B**) Fresh pods weight (g) (FWP) vs Yield, (**C**) Hundred seed weight (g) (HSW) vs Yield, (**D**) Dry pods weight (g) (DPW) vs Yield.

The correlations among the 15 yield related components over the combined analysis are shown in Table 8. The total number of pods showed a strong significant positive correlation with mature pods (r = 0.92), Moderately associated with yield (r = 75), fresh pod weight (r = 78), and dry pods weight (r = 0.75). Moreover, hundred seed weight (r = 0.63) and dry seed weight (r = 0.67) showed a moderate association with total number of pods. The yield per hectare had a positively strong significant association with number of mature pods (r = 0.83), fresh pod weight (r = 0.83), and dry seed weight (r = 0.94). A highly significant and perfect correlation was recorded among dry pods weight with yield (r = 1.00), though, a moderate positive association was found with hundred seed weight (r = 0.84) and harvest index (r = 0.36). Pod width (r = 0.48) and pod length (r = 0.58) showed a considerable degree of association with yield per hectare. A moderate positive association was observed for seed width (r = 0.39) and seed length (r = 0.74) with yield. Figure 1 showed the color map on a cluster of phenotypic traits whereas Fig. 2 depicted a graphical portrayal of the relationship between yield and its strongly contributing factors. In this color map red, and yellow colors indicate the negative and positive correlation of the tested traits, respectively and the intensity of color implies the magnitude of association among the traits. From the color map, we established that the traits such as the total number of pods, fresh pods weight, dry pods weight, yield, dry seeds weight, and hundred seeds weight captured the high intense yellow color indicating that a significant relationship was present among these traits. The yield kg per hectare was directly derived from dry pods weight per plant (g) and we observed a direct linear relationship between yield and dry pods weight (Fig. 2).

### Variance component analysis

Variation in every population is caused by genetics and environmental influences. Only genetic variability is heritable from generation to generation, however, distinguishing between heritable and non-heritable characteristics complicates the selection process for breeders. As a result, before beginning a prudent breeding effort, breeders must differentiate the heritable from the non-heritable variation. Table 9 shows the estimated phenotypic coefficient of variation (PCV) and genotypic coefficient of variation (GCV) for all characteristics. We considered genotypic variance (**σ**^**2**^_**g**_**),** genotype by season variance (**σ**^**2**^_**gs**_), genotype by location variance (**σ**^**2**^_**gl**_), genotype by location by season variance (**σ**^**2**^_**gls**_), and error variance (**σ**^**2**^_**e**_) as variance components which collectively contribute to phenotypic variance (**σ**^**2**^_**p**_). However, the more the variance component lesser the value of heritability and genetic advance^26^. The PCV spanned from 5.2% for days to maturity to 25.91% for biomass dry weight (g) while, the GCV ranged from 1.15% for shelling percent to 24.57% for biomass dry weight (g). Traits such as number of petiole, number of leaves, biomass fresh and dry weight (g) all had high PCV values of more than 20% whereas, days to emergence, days to 50% flowering (d), number of branches, number of stem, number of nodes, internode length (mm), pod length, pod width, seed length, and seed width all had moderate PCV values ranged from 10 to 20% (Table 9). We observed low genotypic coefficient of variation (GCV) for most of the evaluated traits excluding biomass fresh (GCV = 20.11%) and dry weight (GCV = 24.57%) which is greater than 20%.

**Table 9:** Estimation of variance component, heritability, and genetic advance for 27 phenotypic traits in 30 Bambara groundnut accessions.

Trait	Mean	σ^2^_g_	σ^2^_ge_	σ^2^_e_	σ^2^_p_	PCV (%)	GCV (%)	h^2^_B_ (%)	GA (%)
DTE	11.4	1.17	0.91	2.61	4.69	18.99	9.48	24.95	9.76
D50%F	38.96	7.62	9.19	5.12	21.92	12.02	7.08	34.76	8.6
DTM	132.76	15.88	21.38	10.47	47.72	5.2	3	33.27	3.57
PH	29.5	0.89	3.05	4.48	8.42	9.84	3.19	10.53	2.14
NB	38.8	4.85	11.21	17.59	33.64	14.95	5.68	14.41	4.44
NS	18.75	1.31	6.23	2.38	9.92	16.79	6.1	13.18	4.56
NP	303.57	817.48	2968.8	469.73	4256.01	21.49	9.42	19.21	8.5
NL	910.71	7357.3	26,719.1	4227.6	38,304	21.49	9.42	19.21	8.5
NNS	13.78	0.19	1.07	3.21	4.47	15.35	3.16	4.23	1.34
IL	3.51	0.06	0.04	0.19	0.28	15.2	6.7	19.44	6.09
BFW	453.85	8333	1151.6	420.23	9904.83	21.93	20.11	84.13	38
BDW	252.18	3840.2	246.26	183.73	4270.19	25.91	24.57	89.93	48
TNP	84.68	12.45	17.2	19.44	49.09	8.27	4.17	25.36	4.32
NMP	70.69	14.08	15.68	18.07	47.83	9.78	5.31	29.43	5.93
NIP	13.99	2.34	1.5	3.94	7.78	19.93	10.93	30.08	12.35
FPW	633.62	1038.8	2131.7	256.82	3427.32	9.24	5.09	30.31	5.77
DPW	395.57	504.86	14.29	127.54	646.69	6.43	5.68	78.07	10.34
PL	35.17	2.61	14.07	12.14	28.82	15.27	4.6	9.07	2.85
PW	18.09	0.02	1.93	2.51	4.46	11.68	0.84	0.52	0.12
NSP	123.35	36.1	2.17	31.82	70.09	6.79	4.87	51.5	7.2
DSW	305.5	314.41	48.31	275.91	638.63	8.27	5.8	49.23	8.39
SL	17.37	0.33	1.56	2.16	4.05	11.59	3.32	8.18	1.95
SW	12.94	0.3	1.55	1.29	3.15	13.7	4.21	9.46	2.67
HSW	176.33	145.06	24.77	130.22	300.04	9.82	6.83	48.35	9.78
Shell%	77.47	0.79	1.71	17.34	19.84	5.75	1.15	3.98	0.47
HI	62.25	33.27	0.71	2.17	36.15	9.66	9.27	92.05	18.31
YLD	2355	17,887.3	506.35	4519.1	22,912.75	6.43	5.68	78.07	10.34

σ^2^_g_ genotypic variance, σ^2^_ge_ genotype by environment variance, σ^2^_e_ error variance, σ^2^_p_ phenotypic variance, *PCV* Phenotypic coefficients of variation, *GCV* Genotypic coefficients of variation, h^2^_*B*_ heritability, and *GA*  genetic advance; *DTE* Days to emergence (d), *D50%F* Days to 50% flowering (d), *DTM* Days to maturity (d), *PH* Plant height (cm), *NB* Number of branches per plant, *NS* Number of stems per plant, *NP* Number of petioles per plant, *NL* Number of leaves per plant, *NNS* No. of nodes per stem, *IL* Inter nodes length (cm), *BFW* Biomass fresh weight per plant (g), *BDW* Biomass dry weight per plant (g), *TNP* Total no. of pods per plant, *NMP* Number of mature pods per plant, *NIP* Number of Immature pods per plant, *FPW* Fresh pods weight (g), *DPW* Dry pods weight (g), *PL* Pod length (mm), *PW* Pod width (mm), *NSP* Number of seeds per plant, *DSW* Dry seed weight per Plant (g), *SL* Seed length (mm), *SW* Seed width (mm), *HSW* hundred seed weight (g), *Shell %* Shelling percent, *HI* Harvest index (%) and *Yld* Yield (Kg/ha).

**Figure 3:**
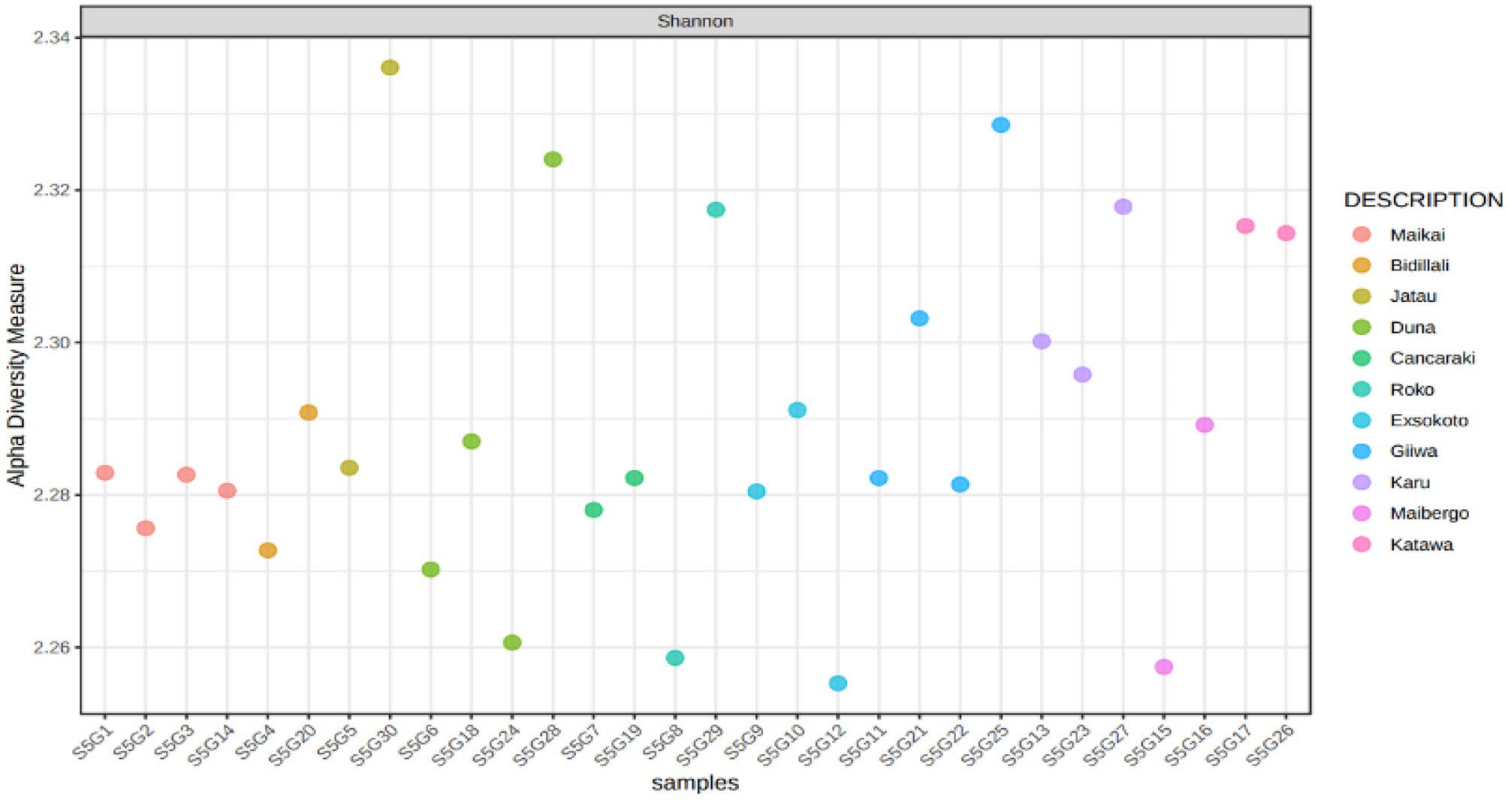
Estimation of Shannon-diversity of 30 evaluated accessions. In the figure right side legend (description indicated the 11 population of Bambara groundnut from which the 30 accessions were sampled. Samples are Duna (S5G6, S5G18, S5G24, S5G28); Maikai (S5G1, S5G2, S5G3, S5G14); Cancaraki (S5G7, S5G19); Roko (S5G8, S5G29); Bidillali (S5G4, S5G20); Jatau (S5G5, S5G30); Maibergo (S5G15, S5G16); Katawa (S5G17, S5G26); Giiwa (S5G11, S5G21, S5G22, S5G25); Karu (S5G13, S5G23, S5G27); and Exsokoto (S5G9, S5G10, S5G12).

Variation in traits was considered for the selection program, which is based on heredity. The assessment of genetic advance with heritability may be a significant tool in crop improvement for determining the expected benefit from the selection. The variables in this investigation indicated low to high heritability estimates ranging from 3.98% for shelling percent to harvest index (92.05%) (Table 9). The broad-sense heritability of biomass dry weight (89.93%), biomass fresh weight (84.13%), dry pod weight (78.07%), harvest index (92.05%), and yield (78.07%) was found to be highly heritable. A moderate heritability was recorded for days to 50% flowering (34.75%), day to maturity (33.27%), number of seed (51.5%), dry seed weight (49.23%) and hundred seed weight (48.35%) (Table 9) remaining of the traits had low heredity. Genetic advance is a measure of how far a population may go via selection. Because heritability does not always imply high genetic gain, but it does when combined with high genetic advance. Vegetative and yield component characteristics have genetic advance values ranging from low to moderate (Table 9). The maximum genetic gain was discovered in biomass dry weight (g), which was 48%, followed by biomass fresh weight (g) (38%), whereas moderate genetic gain was recorded for the traits such as number of immature pod (12.35%), harvest index (18.31%), dry pod weight (10.34%), hundred seed weight (9.78%), and yield (10.34%) (Table 9). These characteristics of moderate to high heritability along with genetic gain have the potentiality of successful selection in genetic improvement. Because their expressions are controlled by additive gene action, the simple phenotypic selection is enough to enhance the next generation. The understanding of the extent and nature of variability across genotypes for certain traits is a requirement for doing simultaneous selection on more traits for Bambara groundnut development. We also estimated the Shannon diversity index (Fig. 3) to explore the extent of diversity, the genotype S5G30 showed a maximum value of more than 2.33 followed by S5G25 close to 2.33 (Fig. 3).

### Clustering pattern

**Figure 4:**
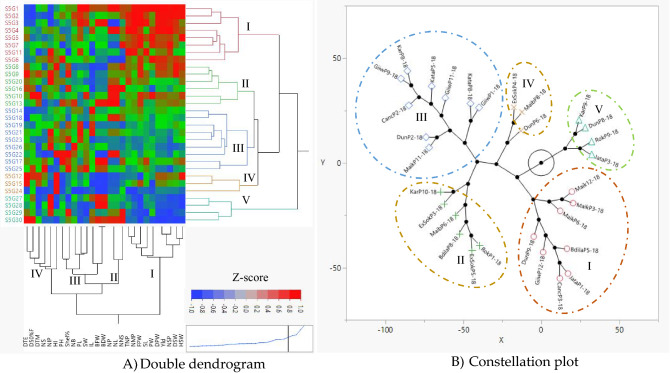
Cluster analysis: (**A**) double dendrogram and (**B**) constellation plot illustrating of phylogenetic relationship of *V. subterranea* genotypes (Cluster I, II, III, IV, and V) represent accessions as indigo, green, blue, and orange symbols.

Genetic differentiation analysis is one of the standard statistics for parental selection, which reveals the degree of divergence across existing genotypes. The clustering provides a very strong and compact indication of the degree and shape of genetic variation, which is important for selecting the expected genotype. The phenotypic data were used in this study to compute the phylogenetic relationship among the 30 Bambara groundnut genotypes. The Ward hierarchical cluster analysis illustrated a two-way dendrogram (Fig. 4A), found distinct clusters indicating relationships among tested genotypes. The two-way dendrogram constructed a double dendrogram at the same plot. The horizontal dendrogram represents the dendrogram for genotypes and the vertical one represents the dendrogram for variables. The cluster I loaded the eight genotypes such as S5G1, S5G3, S5G2, S5G4, S5G5, S5G6, S5G7, and S5G11. The six genotypes viz*.* S5G20, S5G8, S5G9, S5G16, S5G10, and S5G13 were comprised cluster II. The maximum number of genotypes (9) were assembled in cluster III while the minimum genotypes (3) were in cluster IV. However, cluster V had four accessions. In the vertical dendrogram, among the 27 traits, ten traits such as TNP, NMP, FPW, SL, PW, DPW, Yield, NSP, DSW, and HSW into cluster I. Cluster II possesses the three vegetative traits such as NNS, NP, and NL whereas eight variables are grouped into cluster III (BDW, BFW, IL, SW, PL, NB, Shell%, and PH). However, cluster IV assembled the traits like DTE, D50%F, DTM, NS, NIP, and HI. This result had validation based on a correlation test i.e., there was a meaningful relationship among the traits. A heatmap with the cluster represents the chromatic visualization of the relationship between traits and genotypes. In the heatmap, each bar represents the position of accessions and variables intersection point. According to Z-score red and blue colors represent the high and low abundance of traits with accessions. The intensity of the color indicates the magnitude of traits abundance or richness, hence, the more color intensity the more abundance of the components. A constellation diagram (Fig. 4B) showing the correct position of the point representing each accession. Cluster analysis depicting constellation plot of Bambara groundnut (Clusters I, II, III, IV, and V) represent accessions as the indigo circle, red cross, blue square bar, green cross, and pink triangle symbols, respectively.

### Principal component analysis

**Figure 5:**
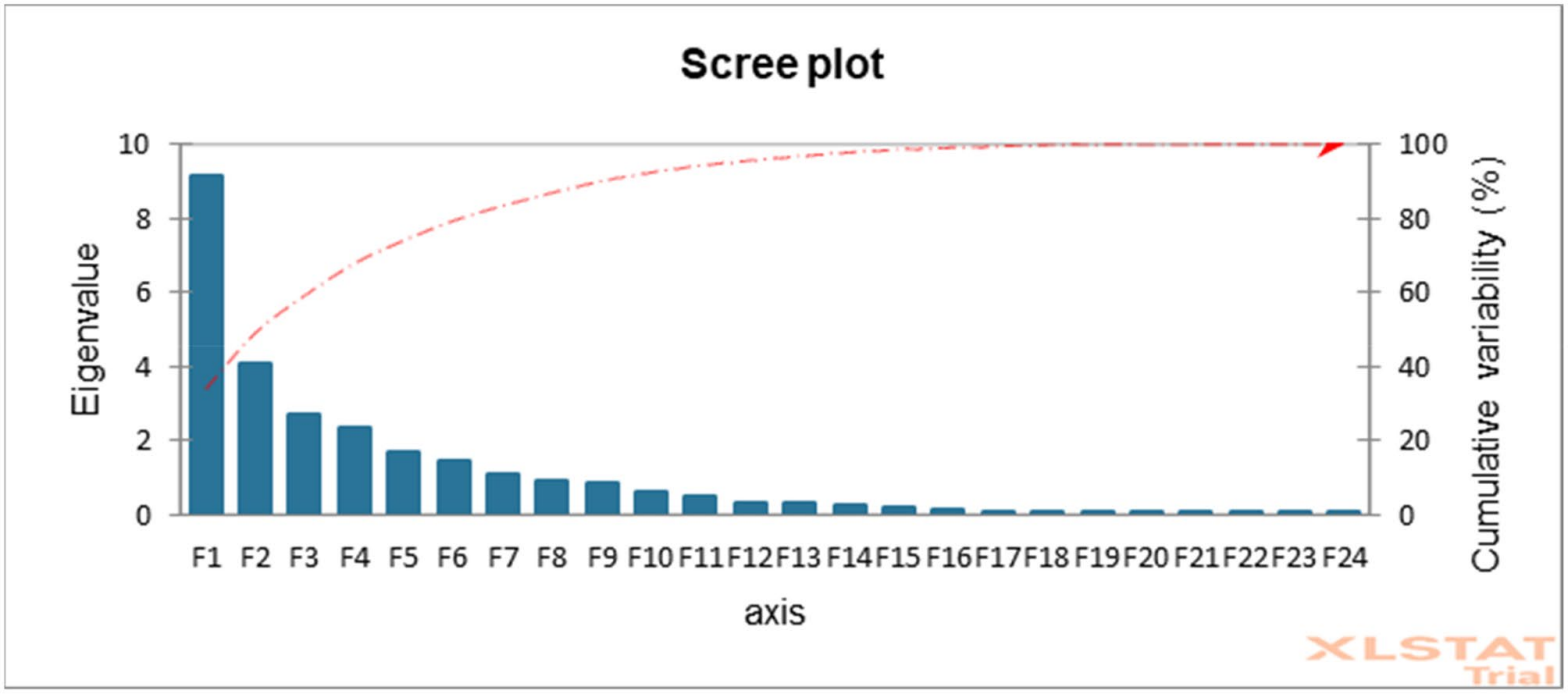
Graphical illustration of eigen values, axis, and % cumulative variation revealed by XLSTAT.

**Figure 6:**
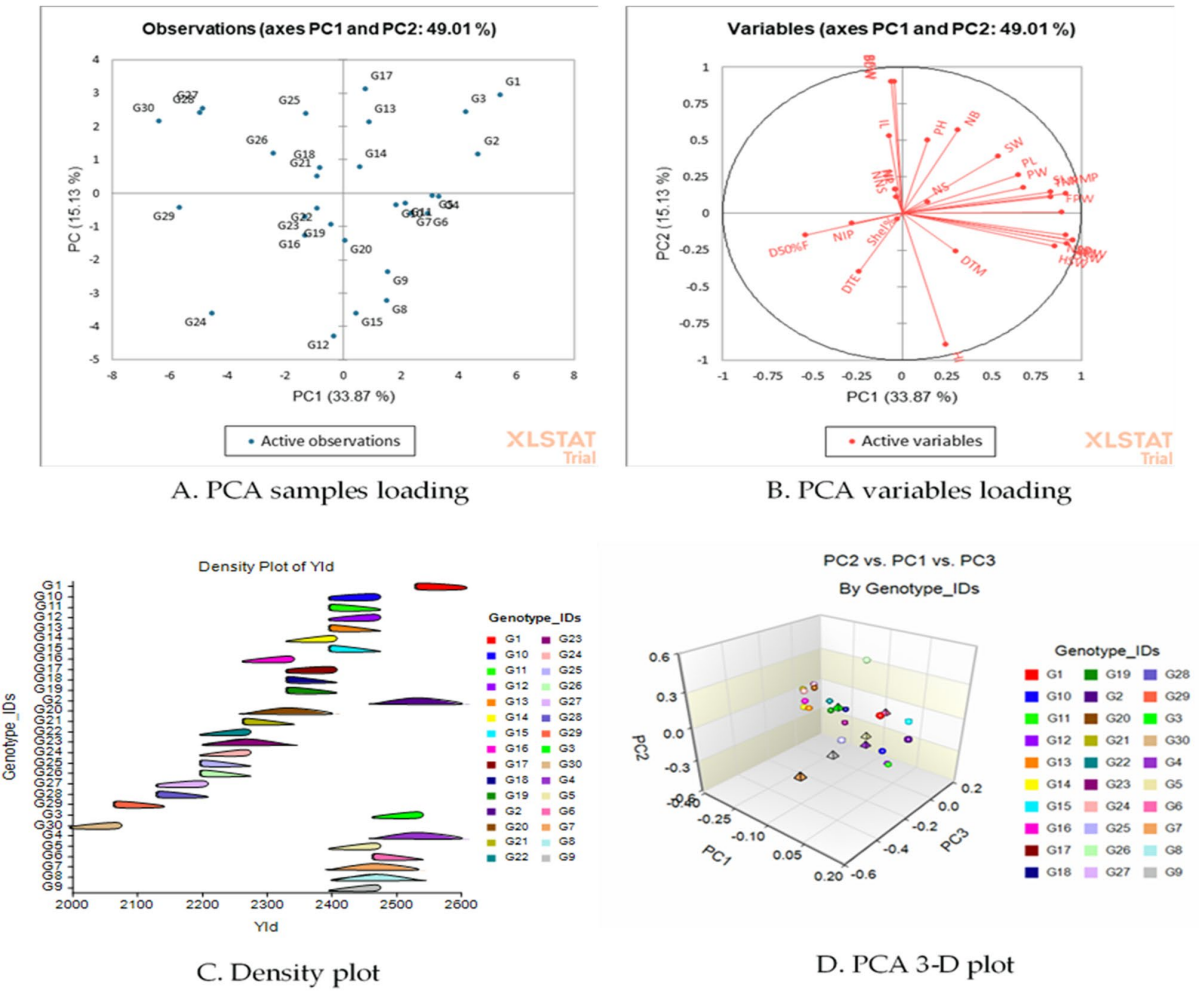
The PCA analysis depicts the (**A**) loading of samples (genotypes) and (**B**) loading of variables (traits) (**C**) density plot showing the genotypes distribution based on yield potential and (**D**) PCA 3D plot revealed by XLSTAT and NCSS 2021.

For sorting characteristics and categorizing accessions, principal component analysis (PCA) has been widely utilized in agricultural research. In the current study, the first seven principal components (PC) accounted for 83.38% of the total variance (Table 10 and Fig. 5). The first PC was gained and recorded for the greatest proportion of variance in the set of all PCs, while the rest were acquired and recorded for decreasingly lower and smaller amounts of variation. The proportion of variance for PC1 and PC2 were 33.87% and 15.13%, respectively, while the 7th PC accounted for 4.07% of the variation. As revealed by an analysis, the traits contributing to PC1 and PC2 have the most variability, with a high coefficient of variation. Table 10 shows the factor loading of several traits that were discovered using principal component analysis. The PC1 allowed loading of most of the traits evaluated indicating the positively significant for the respective principal components except DTE (− 0.08), D50%F (− 0.18), NP (− 0.01), NL (− 0.01), NNS (− 0.01), BDW − 0.01), SHEL% (− 0.01), NIP (− 0.09), IL (− 0.02), and BFW (− 0.02). Simultaneously, for PC2 the traits such as plant height, branch number, total number of pods, biomass fresh and dry weight, pod length, seed width showed significance to the respective principal components. Figure 6A depicts a graphical representation of sample loading (30 accessions) and Fig. 6B variable loadings (27 morphological characteristics) revealed by principal component analysis (PCA). In PCA variable loading, all the traits were positioned on the positive side excluding the traits D50%F, NIP, Shell%, and DTE. The angle between two vectors indicates the degree of association of the respective traits. The lower the angle higher the relationship between the trait and vice-versa. The angle of 90° between the trait indicated no association while more than 90° is suggested as a negative relationship. However, the density plot (Fig. 6C) showed the intensity of the genotypes’ dispersion based on yield potentials. Figure 6D illustrated the three-dimensional (3D) visualization of the 30 evaluated accessions.

**Table 10:** Eigenvalues, variation percentage, and eigenvectors revealed by PCA analysis.

Parameters	PC1	PC2	PC3	PC4	PC5	PC6	PC7
Eigenvalue	9.15	4.09	2.73	2.34	1.68	1.43	1.10
Variability (%)	33.87	15.13	10.12	8.65	6.24	5.30	4.07
Cumulative variance%	33.87	49.01	59.12	67.77	74.01	79.31	83.38
DTE	− 0.08	− 0.20	0.19	0.39	0.00	0.08	− 0.15
D50%F	− 0.18	− 0.07	0.19	0.42	− 0.10	0.07	0.18
DTM	0.10	− 0.13	0.15	0.41	0.05	0.06	0.40
PH	0.05	0.25	0.05	− 0.06	0.25	0.49	− 0.06
NB	0.10	0.28	− 0.06	0.04	0.22	0.17	0.29
NS	0.05	0.04	− 0.10	0.43	0.19	− 0.19	− 0.40
NP	− 0.01	0.08	0.55	− 0.13	− 0.07	0.09	0.12
NL	− 0.01	0.08	0.55	− 0.13	− 0.07	0.09	0.12
NNS	− 0.01	0.06	0.36	− 0.07	− 0.07	− 0.49	− 0.32
IL	− 0.02	0.26	0.05	− 0.28	0.10	− 0.07	0.11
BFW	− 0.02	0.44	− 0.01	0.19	− 0.16	− 0.12	0.00
BDW	− 0.01	0.44	− 0.02	0.18	− 0.15	− 0.12	− 0.01
TNP	0.28	0.06	0.08	− 0.01	− 0.28	0.16	− 0.17
NMP	0.30	0.07	0.07	− 0.04	− 0.12	− 0.05	− 0.01
NIP	− 0.09	− 0.03	0.01	0.07	− 0.39	0.52	− 0.40
FPW	0.30	0.00	0.08	− 0.01	− 0.02	− 0.08	− 0.06
DPW	0.32	− 0.09	− 0.02	0.01	− 0.08	− 0.08	0.08
PL	0.21	0.13	− 0.09	− 0.15	0.20	0.10	− 0.24
PW	0.22	0.09	0.01	− 0.03	− 0.11	0.14	− 0.11
NSP	0.30	− 0.07	0.00	0.01	− 0.20	− 0.11	0.09
DSW	0.30	− 0.10	0.07	0.02	0.10	− 0.06	− 0.05
SL	0.27	0.07	− 0.07	0.16	− 0.05	0.09	0.03
SW	0.18	0.19	− 0.02	0.19	0.30	0.01	0.11
HSW	0.28	− 0.11	0.10	0.07	0.11	0.11	− 0.05
Shel%	− 0.01	− 0.02	0.31	0.04	0.53	0.02	− 0.30
HI	0.08	− 0.44	0.00	− 0.16	0.14	0.08	0.06
Yld	0.32	− 0.09	− 0.02	0.01	− 0.08	− 0.08	0.08

*PC* Principal component, *DTE* Days to emergence (d), *D50%F* Days to 50% flowering (d), *DTM* Days to maturity (d), *PH* Plant height (cm), *NB* Number of branches per plant, *NS* Number of stems per plant, *NP* Number of petioles per plant, *NL* Number of leaves per plant, *NNS* No. of nodes per stem, *IL* Inter nodes length (cm), *BFW* Biomass fresh weight per plant (g), *BDW* Biomass dry weight per plant (g), *TNP* Total no. of pods per plant, *NMP* Number of mature pods per plant, *NIP* Number of Immature pods per plant, *FPW* Fresh pods weight (g), *DPW* Dry pods weight (g), *PL* Pod length (mm), *PW* Pod width (mm), *NSP* Number of seeds per plant, *DSW* Dry seed weight per Plant (g), *SL* Seed length (mm), *SW* Seed width (mm), *HSW* hundred seed weight (g), *Shel%* Shelling percent, *HI* Harvest index (%) and *Yld* Yield (Kg/ha).

## Discussion

### Analysis of variance and mean performance for vegetative and yield components

A significant variation presents in the pooled analysis of variance for the 27 traits of the 30 Bambara groundnut genotypes. Among the vegetative trait, the coefficient of variation ranged from 5.43% for days to maturity to 27.96% for biomass fresh weight (g) however, for the yield component it varied from 6.17% (shelling percent) to 26.24% (hundred seed weight). Masindeni^26^, reported CV = 24.79% for grain yield, CV = 9.32% for hundred seed weight and CV = 31.86% for pods number per plant over six locations. In terms of grain yield and hundred seed weight, our findings revealed highly significant (p ≤ 0.01) variations across genotypes. For almost all characteristics, including dry pod weight, hundred seed weight, and dry seed weight, the G × E interaction was highly significant (p ≤ 0.01), though statistically shelling percent had no meaningful difference. This implies that the genotypes' ordering was not consistent. Similar findings of significance of G × E have been reported by Masindeni^26^, in Bambara groundnut, Oladosu et al.^15^ in rice, and Ali et al.^27^ in groundnut. . In this current research, a significant level of divergence among GE interaction and genotype effect indicated that certain of the presence of diverse multi-environments with different genotypes as well as high yield potential^28^. According to Yan et al.^29^, the GEI over a wide extent of mega- environmental trail comprises of two interactions namely, crossover and non-crossover interactions. The yield performance among the tested accessions over multi-environments persistently designates as non-crossover interaction whereas crossover interaction illustrates the comparative alternation in genotypes ranking over a wide range of environments. Plant breeders can either select genotypes for a certain environment or widely adjusted genotypes across the multi-environmental conditions when GEI is subjected to the influence of predictable components^30^. However, the generation of comparatively constant and stable genotypes over multi-environmental situations is obligatory when GEI is subjected to an unpredictable component^31^ and for well explanation and realization of GEI, yield stability analysis under multi-location and seasons may prompt both reproducibility and heritability of the traits evaluated^32^.

### Estimation of the relationship between traits

Consideration of the correlation matrix might be a fantastic scale of assessment for improved genotype selection programs^33^. Mohammed^34^ supports the use of correlation matrices in plant breeding as a popular method for determining the degree of relationship between two or more traits. This conclusion is similar to the findings of Pranesh et al.^35^ and Jonah et al.^36^ who found a strong and positive significant association between the total number of pods and the attributes such as mature pods number, dry pods weight, seeds number, dry seeds weight, and yield. We found a weak to intermediate and positive relationship between plant height (PH) and yield, biomass fresh and dry weight, and we propose selection based on these qualities may be beneficial for yield increase as well as fodder production for animal feeding. Our findings were corroborated with the research report stated by Mohammed^34^ in Cote d’ Ivoire and Zenabou et al.^37^ in Cameroon on Bambara groundnut. Zenabou et al.^38^ reported days to 50% flowering had a negative correlation with fresh and dry pod weight, which was consistent with our findings due to less variation in planting materials. Plant height was negatiively associated with yield but it was positively associated with shelling percentage, hundred seed weight, fresh pod weight, and these findings agree with the statement of Ahmad^39^. The number of petiole and leaves per plant expressed a positive correlation with total number of pods, fresh pods weight, dry seed weight, hundred seed weight and shelling percent but a negative correlation with harvest index, yield, a similar statement was noted by Unigwe et al.^40^. The yield components such as the total number of pods, fresh and dry pods weight , number of seeds, dry seeds weight, hundred seed weight, and harvest index expressed a positively significant correlation with grain yield in our investigation. These results were comparable with the report on Bambara groundnut, published by Khan et al.^8^, Mohammed^34^, Pranesh et al.^35^, Khan et al.^41^, and Onwubiko et al.^42^. This suggests that these characteristics might be chosen for to increase Bambara groundnut production.

### Variance component analysis

The values of phenotypic variance is greater than the other components studied namely, genotypic variance, genotype by environment variance etc. for all traits, similar to the findings of earlier reports by Khan et al.^8,25^ indicating that trait expression is governed by the environment. According to Sivasubramanian and Madhavamenon^22^, the proportion of GCV and PCV values is deemed low when the value ranges between 0 and 10%, moderate when the value ranges between 10–20%, and high when the value ranges over 20%. However, because the coefficient of variation is independent of the measurement unit, it is more trustworthy when comparing trials^25^. The selection may be beneficial to the traits with lower PCV with higher GCV levels to isolate promising cultivars. On Bambara groundnut, Onwubiko et al.^43^, Gonné et al.^44^, and Naik^45^ reported similar findings, as well as in groundnut reported by Ambros^46^. According to earlier findings, selection may be successful for a specific trait improvement by effectively utilizing genetic variation with the higher degree of heritability^47,48^. In a broad sense, heritability refers to the fraction of total variation in phenotypic variables across individuals in a particular group that may be attributed to genetic variation. Higher genotypic coefficient of variation coupled with high heritability as well as high genetic advance gives better clues than the individual measures of variance component^49^. According to Johnson et al.^24^, the heritability percentage is deemed low when the value runs between 0 and 30%, moderate when the value runs between 30 and 60%, and high when the value exceeds 60%. Breeders might use high heritability for certain characteristics to choose superior genotypes based on phenotypic observations^43,48^. Low heritability, on the other hand, denotes a low heritable component of variation and a greater influence of environmental effects on the expression of such trait, as a result, selection based on such characteristics is futile, according to Ridzuan et al.^50^. Jaiswal et al.^51^ on the other hand, emphasized that attributes linked with high heritability do not always result in a higher genetic advance; hence, high heritability coupled with high genetic advance gives a more credible outcome. Furthermore, heredity encompasses both additive and non-additive gene action; hence, heritability should be considered in conjunction with genetic advancement for predicting the selection of superior genotypes^52^. The proportion of genetic advance is deemed modest, with values ranging from 0 to 10%, moderate (10–20%), and high (> 20%)^24^. However, in our investigation moderate to high heritability was identified in yield components coupled with moderate to high genetic advance and this is the evidence of medium to high environmental influences on these traits. A similar observation was reported by Masindeni^26^ and Khaliqi et al.^9^ in Bambara groundnut. However, using low to medium heritable traits, improvement in the following generation may not provide the expected outcomes since it has been proposed that non-additive gene action i.e., epistatic and the interaction between genotype and environment play a substantial influence in the expression of this trait^26^. Depending on the variability and heritability estimations, it is possible to deduce that direct selection can improve variables such as hundred seed weight (g), harvest index, biomass fresh and dry weight (g), number of seed, dry seed weight, and yield per hectare in Bambara groundnut. High heritability and genetic advance observed high in biomass fresh weight and biomass dry weight is the representing traits of yield per hectare is supported by Molosiwa^49^. We recorded Shannon diversity index range from 2.25 to 2.34 indicating that the evaluated genotypes showed a significant level of variation over the environment. Similar findings have been noted by Khan et al.^7^, who stated a standard scale for Shannon diversity value of 1.5 to 3.5.

### Clustering pattern and PCA analysis

Based on the heatmap we observed that the genotypes under clusters I and II captured more red hue with the association of the yield and its contributing traits under cluster I. Our findings were advocated by Khan et al.^7,8^ in Bambara groundnut. Several analyses using various agglomeration approaches were tried to obtain the best possible categorization of accessions. The Ward technique seems to be the greatest agglomeration method for producing the finest results^53^. The constellation plot (Fig. 4B) arranges the accessions as endpoints and each cluster joins as a new point, with lines drawn that represent the membership. The wider the lines, the higher the distance between groups. The current clustering investigation was supported by previous research, which was noticed by Unigwe et al.^40^, and Bonny et al.^54^ found substantial variation in morphological features of Bambara groundnut. Moreover, Kumari et al.^53^ in Maize, Doumbia et al.^55^ in cowpea. In PCA sample loading, except the accessions, S5G12, S5G24, S5G19, S5G16, S5G23, S5G22, and S5G29 rest of the genotypes were palaced into positive parts of the PCA plot. These findings are validated by the report of Khan et al.^7,8^. The goal of the principal component analysis is to identify the total variance in a group of characteristics that successively accounts for the most variability in the data^50^. In general, traits are inter-correlated to various degrees, thus all of the principal components are not necessary to properly summarise the data. In any PCA, the first axes (PC1) explain the greatest proportion of the overall variance^56^. Shegro et al.^57^ used PCA analysis to categorize the 20 Bambara groundnut accessions whereas Mohammed^58^ found that PC1 and PC2 contributed to the overall variation at 19% and 14%, respectively.

## Conclusion

The combined analysis of variance indicated that genotype (G), environments (E) and genotype by location (G × E interaction) showed extremely significant variations in vegetative, yield, and yield component characteristics. According to the means comparison results, Bambara groundnut genotypes G1, G3, G5, G6, G8, G7, G2, G4, G10, G13, G11, and G14 were the closest to the ideal genotype with superior yield across the environment. These genotypes were grouped into similar clustering according to Ward hierarchical clustering methods which assembled the accessions into five distinct clusters. Considering the pooled data PCA accounted for 49.01% variation contributed by PC1 (33.87%) and PC2 (15.13%). Farmers will reap high yields and steady revenue if better genotypes with the capacity to give a consistently high yield over different conditions are identified and certified for cropping. According to the results shown above, the planting materials have a sufficient level of genetic variation. This indicates that there is enough diversity to be exploited by selection. As a result, greater GCV, broad-sense heritability, and genetic advance are demonstrated by the various yield component features, particularly dry pods weight, hundred seed weight, biomass fresh and dry weight, dry seed weight, harvest index, total number of pods significantly impact the yield. As a result, they would be receptive to positive selection. In deciding yield and yield components, the environment played a greater role than genotype, and G × E interaction accounted for significant variation resulted, complicating genotype selection, an additional statistical analysis is necessary to estimate the stability of each genotype throughout the whole environment. The application of stability statistical measures is recommended to analyze genotype stability that divulges several G × E interaction features, resulting in the detection of stable genotypes across environments. However, statistical techniques such as univariate and multivariate analysis can be more fruitful in unfolding and understanding the G × E interaction alongside variance component analysis. Overall, this result will assist plant breeders in this crop improvement as well as selecting superior lines for the future breeding program.

## Data Availability

All data generated or analyzed during this study are included in this published article.
